# Perturbation of hyperthermia resistance in gastric cancer by hyperstimulation of autophagy using artemisinin-protected iron-oxide nanoparticles†

**DOI:** 10.1039/d4ra05611f

**Published:** 2024-10-29

**Authors:** Komal Attri, Bhupendra Chudasama, Roop L. Mahajan, Diptiman Choudhury

**Affiliations:** a Department of Chemistry and Biochemistry, Thapar Institute of Engineering and Technology Patiala 147004 Punjab India diptiman@thapar.edu +91-8196949843; b Department of Physics and Material Sciences, Thapar Institute of Engineering and Technology Patiala 147004 Punjab India bnchudasama@thapar.edu +91-9781966136; c Department of Mechanical Engineering, Department of Materials Science & Engineering, Virginia Tech Blacksburg VA 24061 USA mahajanr@vt.edu +1-5402312597; d TIET-VT Centre of Excellence for Emerging Materials, Thapar Institute of Engineering and Technology Patiala 147004 Punjab India

## Abstract

In a bid to overcome hyperthermia resistance, a major obstacle in cancer treatment, this study explores manipulating autophagy, a cellular recycling mechanism, within the context of gastric cancer. We designed artemisinin-protected magnetic iron-oxide nanoparticles (ART-MNPs) to hyperactivate autophagy, potentially sensitizing cancer cells to hyperthermia. The synthesized ART-MNPs exhibited magnetic properties and the capability of raising the temperature by 7 °C at 580.3 kHz. Importantly, ART-MNPs displayed significant cytotoxicity against human gastric cancer cells (AGS), with an IC50 value of 1.9 μg mL^−1^, demonstrating synergistic effects compared to either MNPs or ART treatment alone (IC50 for MNPs is 9.7 μg mL^−1^ and for ART is 9.4 μg mL^−1^ respectively). Combination index studies further supported this synergy. Mechanistic analysis revealed a significant increase in autophagy level (13.58- and 15.08-fold increase compared to artemisinin and MNPs, respectively) upon ART-MNP treatment, suggesting that this hyperactivation is responsible for hyperthermia sensitization and minimized resistance (as evidenced by changes in viability compared to control under hyperthermic conditions). This work offers a promising strategy to modulate autophagy and overcome hyperthermia resistance, paving the way for developing hyperthermia as a standalone therapy for gastric cancer.

## Abstract 2

In a bid to overcome hyperthermia resistance, a major obstacle in cancer treatment, this study explores manipulating autophagy, a cellular recycling mechanism, within the context of gastric cancer.

## Graphical abstract

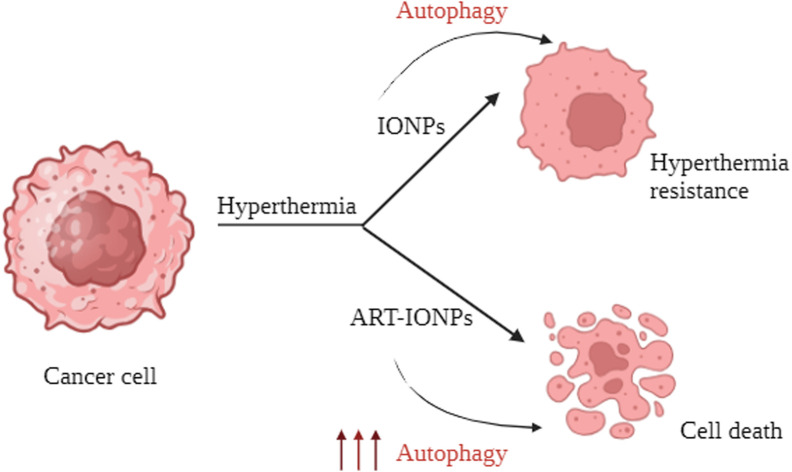

## 1. Introduction

Despite advancements in cancer diagnosis and treatment strategies, cancer remains a leading cause of mortality worldwide.^1,2^ Among the diverse range of cancer types, gastric cancer ranks as the fifth most prevalent cancer worldwide and is the third leading cause of cancer death globally.^3^ Factors that increase the likelihood of developing the condition and influence the treatment outcome include *Helicobacter pylori* infection, genetics, stage of detection, advancing age, consuming excessive amounts of salt, chronic inflammation, malnutrition, and lack of dietary fibre consumption, *etc.*^3^ However, existing cancer treatments, including chemotherapy, often exhibit limitations due to non-specific cytotoxicity, leading to immune system suppression and increased risk of secondary infections in patients.^1,4^ The rise of drug resistance has created a greater need for natural agents that can effectively eliminate tumors and minimize the chances of recurrence.^5^

Nature provides a wealth of secondary metabolites that have been used for centuries to treat various diseases, including gastric cancer.^6^ The global research community is actively exploring the potential of natural materials, such as medicinal plants or potent medicinal components, for developing anti-cancer products.^7,8^ Various secondary metabolites, such as carvacrol, geraniol, sageone, carnosic acid, *etc.*, are used to treat gastric cancer.^9^ Among these, artemisinin, a terpene derived from the Chinese herbal medicine *Artemisia annua* L., also known as sweet wormwood,^10^ is widely acknowledged for its diverse range of properties, including anti-inflammatory, anthelmintic, antipyretic, anti-bacterial, insecticidal, and anti-cancer effects.^11–13^

Artemisinin is a remarkable bioactive compound that has captivated the scientific community's attention. It stands out as a unique and compelling drug with significant biological importance. Unlike many synthetic drugs that have undesirable side effects, artemisinin, a natural plant-based compound, holds considerable therapeutic value.^14^ Its structure possesses an endoperoxide moiety capable of reacting with iron to generate cytotoxic free radicals. Recognizing that cancer cells contain noticeably higher intracellular free iron levels compared to normal cells, artemisinin selectively induces apoptosis in cancer cell lines.^15^ Additionally, artemisinin has been found to induce autophagy, a highly conserved cellular degradation process that helps cells adapt to various stressful conditions, such as nutrient scarcity.^16,17^ During the initial phases of autophagy, impaired cytoplasmic components are enclosed within specialized formations called autophagosomes.^18^ In cancer cells such as colorectal cancer, cervical cancer, multiple myeloma, and promyelocytic leukaemia, artemisinin is known to inhibit NF-kβ activity, thereby inducing autophagy.^19^ It also prevents cell growth and blocks the cell cycle. These unique properties make artemisinin a potential candidate for cancer chemotherapeutic drugs.

Artemisinin holds a distinct advantage as an anti-cancer agent, not solely due to its potency in inducing toxicity in cancer cells but also due to its remarkable selectivity in targeting and eliminating them. Furthermore, its impact on normal cells is characterized by minimal toxicity, further highlighting its potential as an effective treatment for cancer.^20^ Recent research findings have suggested the potential of utilizing it as a therapeutic option for gastrointestinal, breast, brain, and colon cancers.^21^ Apart from its anti-cancer activity, artemisinin is known to exhibit anti-bacterial activity against Gram-negative and Gram-positive bacteria by following multiple mechanisms, including disruption of bacterial cell membranes and potential interactions with different cellular factors. Artemisinin is known to produce cytotoxic carbon-centered radicals after being activated by ferrous ions or reduced heme. These radicals are found to target external microbial organisms, ultimately leading to their death.^22,23^ Recent studies have explored the use of magnetic nanoparticles for targeted drug delivery to specific sites using external magnetic fields. This approach helps reduce toxicity to normal cells and tissues. Iron oxide magnetic nanoparticles, known for their physiological inertness and superparamagnetic properties, play a crucial role in accumulating nanoparticles within cancer cells at the desired target site through the assistance of an external magnetic field.^24,25^ These nanoparticles and magnetic fields exhibit compatibility with biological systems, allowing their application to any region of the body. Ranging in size from 1 nm to 1000 nm, these nanoparticles consist of polymers and drugs, enabling their versatile use in various biomedical applications^26,27^ including as a source of hyperthermia in cancer treatment.

Hyperthermia is believed to be an adjuvant therapy for improving the effectiveness of traditional radiotherapy and chemotherapy. However, some challenges remain. A recent study showed that cancer cells become more resistant to higher temperatures than normal cells in a hyperthermic environment. Furthermore, hyperthermia often fails to induce apoptosis in tumor cells, as these cells can resist hyperthermia *via* the non-activation of caspase 3. Additionally, cancer cells treated with hyperthermia exhibit pseudopod-like extensions, which are not observed in their counterparts without hyperthermia treatment.^28^ Along with their cancer-targeting abilities, iron oxide is known to exhibit anti-bacterial potential against multiple drug-resistant bacterial strains by generating free radicals called reactive oxygen species, which cause oxidative stress and ultimately cause cell death.^29^

One approach that has been investigated to address these problems is the deployment of artemisinin-based magnetic nanoparticles for their anti-cancer activity. By utilizing an external magnetic field, these particles can be directed to selectively accumulate at the intended target site. This targeted delivery approach allows for reduced doses to achieve therapeutic concentrations, consequently minimizing the risk of side effects on normal healthy cells that might arise from higher doses.^27^ This targeted delivery and release of artemisinin in a controlled manner can achieve higher anti-cancer activity than free drugs.^30^ Magnetic nanoparticles loaded with artemisinin have shown notable toxicity in breast cancer cells (MCF-7). Under acidic conditions of the tumor microenvironment, they can produce ferrous ions that catalyze artemisinin to produce ROS, resulting in cell death.^31^

Building upon the information provided above, our hypothesis revolves around the idea that the delivery of artemisinin after conjugation with magnetic nanoparticles (ART-MNPs) into the gastric tissue, coupled with subsequent treatment through hyperthermia, could potentially enhance the efficacy of gastric cancer therapy by overcoming the resistance of tumor cells against hyperthermia. Additionally, the anti-bacterial activity of synthesized nano-formulations against the causative agent of gastric cancer, that is, *H. pylori*, could enhance the potential drug efficacy.

## 2. Materials and methods

### 2.1 Materials

Ferric chloride (FeCl_3_), ferrous sulfate (FeSO_4_), polyethylene glycol (PEG 400), sodium hydroxide (NaOH), and deionized water were procured from LobaChemie, India. Artemisinin was obtained from TCI Chemicals. Ethylene dichloride (EDC), *N*-hydroxy succinimide (NHS), HAMs cell culture media, fetal bovine serum (FBS), Brain Heart Infusion (BHI) broth, agar, penicillin–streptomycin, monodansylcadaverine (MDC), and acridine orange (A.O.) were all sourced from HiMedia, India.

### 2.2 Development of artemisinin-conjugated magnetic nanoparticles

Magnetic nanoparticles were formulated *via* a co-precipitation method. Briefly, iron(iii) chloride hexahydrate and iron(ii) sulfate heptahydrate were added to 50 mL of distilled water, followed by heating to 90 °C. Subsequently, 3 mL of polyethylene glycol (PEG400) was added, followed by adding ammonium hydroxide solution obtained by dissolving 10 mL of 25% ammonium hydroxide in 50 mL of water under continuous stirring for 30 minutes. The final mixture, which was black, was centrifuged for 10 minutes at 4000 rpm after being brought to room temperature. Finally, nanoparticles were washed with deionized water five times to get rid of unbound impurities.

After that, the conjugation of nanoparticles was done with artemisinin. A mixture containing 250 μL each of 10 mg mL^−1^ EDC, 10 mg mL^−1^ NHS, and 7 μL of 1 M NaOH was prepared and added into the pre-synthesized magnetic nanoparticles (0.5 mg) for activating surface carboxyl groups under sonication for 15 minutes. Subsequently, 250 μL (3 mg mL^−1^) artemisinin was added, and the solution was kept at 37 °C for 24 h. The obtained solution was centrifuged for 1 h at 12 000 g for purification. Finally, the nanoparticles were dispersed in phosphate-buffered saline (PBS) and stored at 4 °C for further use.

### 2.3 The studies to determine the size and morphology of nanoparticles with elemental analysis

The hydrodynamic size of the nanoparticles was determined using a Malvern DLS-zeta size analyzer. High-Resolution Transmission Electron Microscopy (HR-TEM) (Talos F200S G2, Thermo Scientific) was used to obtain the exact size, shape, and morphology of ART-MNPs. Before analyzing, the centrifugation of nanoparticles was done for 15 minutes at 240 rpm to remove unbound impurities. The final pellet was analyzed using an energy-dispersive X-ray Spectrometer (EDS) (Bruker QUANTAX 200) to determine the elemental composition.

### 2.4 FTIR spectroscopy to monitor interactions between ART and MNPs

The interactions between ART (artemisinin) and MNPs were investigated using Fourier Transform Infrared Spectroscopy (FTIR) to identify the chemical bonds and study the interactions between artemisinin and magnetic nanoparticles. The FTIR analysis was carried out using an Agilent Cary 600 series Spectrophotometer. The pellets were made using the potassium bromide followed by scanning over 400 cm^−1^ to 4000 cm^−1^. FTIR data will provide a detailed study of variation in the frequency range of artemisinin functional groups after binding with magnetic nanoparticles.

### 2.5 XRD analysis

X-ray diffraction (XRD) unveils a material's crystallographic structure, chemical composition, and physical properties. Here, the technique probed the synthesized nanomaterial. After drying to eliminate moisture's influence, the sample was exposed to CuKα radiation (*λ* = 1.54 Å) for diffraction pattern acquisition within a 2*θ* range of 10–90°. The Scherrer equation, applied to the most intense peak in this pattern, then determined the crystallite size of both MNPs and ART-MNPs.1
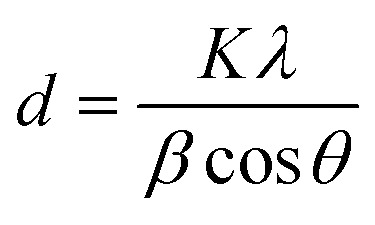
where *K* = 0.9 is the Scherrer constant, *λ* = 1.54 Å denotes the X-ray wavelength, *β* determines the broadening of the highest intense peak, and *d* denotes the crystallite size of the synthesized MNPs and ART-MNPs.

### 2.6 Heating capacity determination using hyperthermia study

In cancer treatment, hyperthermia utilizes elevated cellular temperatures (typically between 42 °C and 45 °C) to target and destroy tumors. To assess the potential of a synthesized material for magnetic hyperthermia applications, we utilized the NanoTherics Magnetherm instrument. This instrument is equipped with an optical fiber temperature probe to precisely record material heating during the process. The prepared sample was exposed to an alternating magnetic field of 10 mT at various frequencies (161.9 kHz, 242.4 kHz, 411.1 kHz, 580.2 kHz, and 935.3 kHz).

### 2.7 Determination of magnetic properties using VSM

To assess the magnetic properties of the synthesized nanoparticles (MNPs and ART-MNPs), we employed a Vibrating Sample Magnetometer (VSM) at room temperature. Before analysis, the samples were dried in powder form. The VSM exposed the samples to a varying magnetic field of ±10 kilo-oersted, allowing us to record the hysteresis loop (M–H), which reveals the relationship between the material's magnetization and the applied magnetic field. This loop is a characteristic of ferromagnetic materials and provides valuable data on the nanoparticles' magnetic properties, including saturation magnetization (*M*_s_), coercivity (*H*_c_), and remanence (*M*_r_).^32^

### 2.8 Studies to determine drug loading and release kinetics

The release profile of artemisinin (ART) from ART-MNPs was investigated through centrifugation and spectrophotometry. For this, 1 mL sample was centrifuged at 10 000 rpm for a duration of 15 minutes to separate the unbound ART in the supernatant from the ART bound to the pellet. The unbound ART concentration in the supernatant was then quantified using spectrophotometric analysis. The pellet was resuspended in 500 mL of water to monitor sustained release. At predetermined time intervals over 40 h, 2 mL aliquots were withdrawn and replaced with fresh water to maintain the original volume. The absorbance of these aliquots was measured at 292 nm, providing information on the ART release pattern at physiological conditions (pH 7 and 37 °C).^15,33,34^ The final data is plotted by taking the mean of three independent experiments.

### 2.9 *In vitro* studies

#### 2.9.1 Cytotoxicity studies

The toxicity of the samples towards cells was evaluated using the MTT (3-(4,5-dimethylthiazol-2-yl)-2,5-diphenyltetrazolium bromide) assay. This assay was performed on AGS cells, a gastric adenocarcinoma cancer cell line. For the experiment, 10 000 cells per well were seeded in a 96-well plate and allowed to reach 75–80% confluency. The cells were then treated with four different concentrations (2.5, 5, 7.5, and 10 μg mL^−1^) of the prepared formulations, including FeCl_3_, FeSO_4_, MNPs, ART, and ART-MNPs. After treating them with formulations, the cells were placed overnight in an incubator at 37 °C. After incubation, the cells were washed with PBS. The MTT solution was then added and incubated for an additional 3 h. The MTT media was removed from each well, and 100 μL of DMSO was added for a 15 minutes incubation. Finally, the absorbance of the resulting solution was measured at 570 nm. The given equation was used to calculate the inhibition percentage.

2 % inhibition = [1 − (*A*_t_/*A*_c_) × 100]%

where *A*_t_ is the test substance absorbance and *A*_c_ is the control solvent absorbance. The experiment was performed three times to get the concordant values.

#### 2.9.2 Scratch assay for anti-cancer activity

A scratch assay was used to determine the anti-cancer activity of the synthesized particles. One 6-well plate was used to grow cells in FBS-free media and placed in an incubator maintained at 37 °C and 5% CO_2_. The plate was then confluent to 80–90%. A sterile 10 μL pipette tip was used to scratch a uniform wound across the cell monolayer carefully. The cells were then washed with PBS to remove debris and impurities. Fresh media was added, and the cells were treated with various samples: FeCl_3_, FeSO_4_, MNPs, ART, and ART-MNPs. Images of the scratch were captured at 6, 12, 24, 36, and 48 h post-treatment.

The scratch width was measured at three random places in all wells at each time point to quantify anti-cancer activity. The average scratch diameter was then calculated, and a graph was plotted to visualize the changes in scratch diameter over time for each sample. This allowed for the assessment of the anti-proliferative effects of the synthesized samples on the cancer cells.^35,36^

### 2.10 Detection of autophagy by monodansylcadaverine (MDC) staining

To assess autophagic activity, AGS cells were cultured in 6-well plates and allowed to reach optimal confluency (70–80%). Each well was then treated with one of the following samples: control, MNPs, ART, or ART-MNPs. Following a 24 h incubation, cells were washed with PBS before trypsinization. The cells were suspended in PBS and treated with 50 μM MDC for 15 minutes at room temperature. Finally, the 1 × 10^4^ cells were analyzed using flow cytometry.

### 2.11 Detection of autophagy by acridine orange (A.O.) staining

An A.O. staining assay was performed to evaluate autophagy similar to the MDC staining procedure described above, AGS cells were grown using 6-well plates till they reached 70–80% confluency. The subsequent steps were identical to those outlined in Section 2.10 above.

### 2.12 Anti-bacterial activity of synthesized ART-MNPs

#### 2.12.1 Antimicrobial activity by determining the minimum inhibitory concentration (MIC)

To assess the antibacterial activity of the nano-formulations, the minimum inhibitory concentration (MIC) was measured, which is the lowest concentration at which the growth of bacteria is effectively inhibited by the anti-bacterial agent over a specified period. The experiment was conducted using *Helicobacter pylori* to determine the MIC of ART-MNPs. Following established procedures, the microdilution was employed to assess anti-bacterial potential in 96-well plates. Initially, bacterial cultures were cultured and maintained in a brain heart infusion (BHI) broth medium in a hypoxia chamber at 37 °C, with 0.1% oxygen and 10% CO_2_ for 24 h. Subsequently, the bacterial suspension was adjusted to a concentration of 10^−8^ CFU mL^−1^ in the BHI medium. For evaluating the anti-bacterial efficacy of the synthesized particles, 20 μL of bacterial suspension, 20 μL of nanoparticles (2.5, 5, 7.5, and 10 μg mL^−1^), and 160 μL of BHI broth were added to each well of the 96-well plates. Negative controls consisted of inoculated broth without nanoparticles. The plate was kept overnight in a hypoxia chamber at 37 °C, in the presence of 0.1% oxygen and 10% CO_2_.^37^ O.D. was taken at 600 nm for the growth analysis.

#### 2.12.2 Anti-bacterial activity by agar well diffusion assay

The agar well diffusion method was used to check the antimicrobial activity of synthesized ART-MNPs. One Petri dish was made using BHI agar for the *H. pylori* strain, a Gram-negative bacterium. A 50 mL solution of BHI media and agar was autoclaved, poured into the Petri plates, and allowed to solidify. After solidification, 5 mm wells were made in the plate with the help of a cork borer, and sterile spreaders were used to spread 50 μL of bacteria on the plate. Three formulations, including ART, MNPS, and ART-MNPs with a concentration of 10 μg mL^−1^, were added to their respective wells and kept for 24 h in a hypoxia chamber at 37 °C, in the presence of 0.1% oxygen and 10% CO_2_. After the incubation period, the inhibition zones around each were observed to assess the antimicrobial efficacy of the formulation.

### 2.13 Determination of combination index (CI) for MNPs and ART

The combination index quantifies the impact of two distinct drugs when used together. This interaction may result in either synergistic or antagonistic effects. Calculating the drug combination index (CI) determines the extent of synergistic or antagonistic effects. A CI value less than 1 indicates synergism, where the combined administration of two drugs enhances each other's activity. Conversely, a CI value exceeding 1 (CI > 1) demonstrates the antagonism and suggests that one drug inhibits the activity of the other drug. A CI value of 1 indicates that neither drug interferes with the other. The CI was determined by computing and assessing the cell viability of AGS cells across different concentrations of ART and MNPs, using eqn (3)3
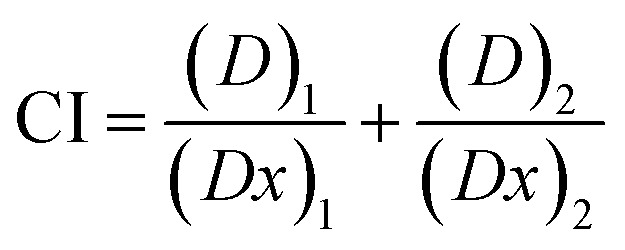
where,

4 *Dx* = *Dm*[*fa*/*fu*]^1/*m*^

Here, (*D*)_1_ and (*D*)_2_ concentrate on ART and MNPs. The concentrations (*Dx*)_1_ and (*Dx*)_2_, indicative of the median effective doses for the individual drugs, are determined through eqn (4), where the affected and unaffected cell fractions in the median dose are denoted by *fa* and *fu* and are equal to 10^(*y*-intercept)/*m*^, where *m* represents the slope median in the median effect plot of log(*D*) *vs.* log(*fa*/*fu*).^38^

## 3. Results and discussions

### 3.1 Structure and composition of ART-MNPs

#### 3.1.1 Shape and size

**Fig. 1:**
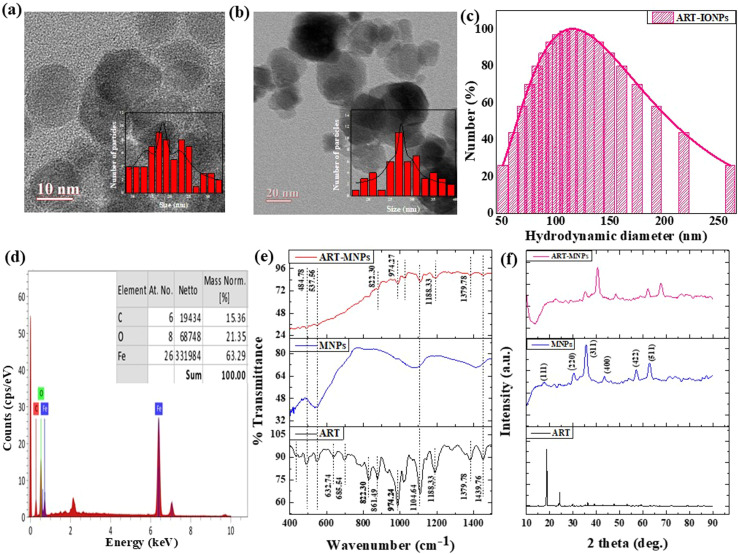
Characterizations of ART-MNPs (a) HR-TEM micrographs at a 10 nm scale reveal the size of IONPS with an inset showing the histogram average diameter of 16 nm (b) HR-TEM micrographs reveal the size of ART-IONPs which comes out to be 26 nm (c) hydrodynamic size of ART-MNPs was analyzed by DLS and the size was found to be 114 nm (d) EDS confirming the presence of iron in ART-MNPs (e) FTIR graph demonstrating the interactions taking place between ART and MNPS (f) XRD plot providing the insights in the crystal structure of ART-MNPs.

Transmission electron microscopy (TEM) images (Fig. 1a) confirmed the spherical morphology of the IONPs with an inset showing the histogram average diameter of 16 nm. Similarly, Fig. 1b shows the TEM images of ART-MNPs, and the histogram inset in this figure shows the average diameter of about 26 nm. The figure used for calculating the average size of IONPs and ART-MNPs is given as ESI data in Fig. S1b and S1c,† respectively. This microscopic analysis was performed at a scale of 20 nm, allowing for detailed observation of the nanoparticle size and shape.

#### 3.1.2 Hydrodynamic diameter

Dynamic light scattering (DLS) measurements revealed a hydrodynamic diameter of 114 nm for the ART-MNPs (Fig. 1c) and 74 nm for IONPs given in Fig. S1a.† This value represents the average size of the nanoparticles when dispersed in solution and accounts for the presence of any solvating molecules or surface functional groups.

#### 3.1.3 Elemental composition

Energy-dispersive X-ray spectroscopy (EDS) analysis (Fig. 1d) confirmed the presence of key elements in the ART-MNPs, including carbon (C), oxygen (O), and iron (Fe). Notably, the iron content was found to be 63.29% and evenly distributed throughout the nanoparticles, indicating the successful incorporation of iron oxide within the ART-MNPs structure.

### 3.2 FTIR for studying interactions between ART and MNPs after nanoparticle synthesis

To explore the interaction between artemisinin ART and MNPs, FTIR was performed on ART, MNPs, and ART-MNPs samples. The findings unveiled multiple peaks in the I.R. spectra, offering valuable insights into the interactions in the examined samples, see Fig. 1e and Table 1. A peak at 537.56 cm^−1^ was identified in MNPs and ART-MNPs, indicating the presence of the Fe–O bond.^41^ Peaks at 632.74 cm^−1^ and 685.53 cm^−1^ were present only in ART, denoting CH_2_ rocking vibrations. Notable peaks at 822.30 cm^−1^ and 861.49 cm^−1^ s identified as C–H bonds of the aromatic C–H^40^ were present in ART but absent in both MNPs and ART-MNPs. A distinct peak, pointing to C–C stretching at 974.27 cm^−1^,^41^ was observed in both ART and ART MNPs but was absent in MNPs. On the other hand, C–O–C asymmetrical stretching vibrations corresponding to peaks at 1104.64 cm^−1^ and 1182.22 cm^−1^ (ref. 42) were noted across all the samples. Furthermore, a peak at 1379.78 cm^−1^ representing C–O vibration was present in ART and ART-MNPs but absent in MNPs. In both ART and ART-MNPs, a bond indicative of C=O stretching was also present at 1439.76 cm^−1^, which was not observed in MNPs.^42^ At 1633.32 cm^−1^, a peak position represented free carboxylic acid in MNPs and ART-MNPs but not in ART.^43^ The –C(=O)–O lactone bond was observed at 1731.70 cm^−1^ in ART and ART-MNPs but is absent in MNPs.^44^ Moreover, a peak indicative of C–H stretching vibrations (alkanes) was observed at 2834.65 cm^−1^ in ART and ART-MNPs but not in MNPs.^39^ Likewise, a bond representing CH_2_ stretching vibrations at 2901.84 cm^−1^ was present in ART and ART-MNPs and absent in MNPs.^40^ O–H stretching vibrations were observed at 3300.15 cm^−1^ in ART-MNPs only.^44^

**Table 1:** The table gives the comparative values of the wavenumbers obtained from the FTIR (in the range of 400–4000 cm^−1^) of artemisinin (ART), IONPs and ART-IONPs indicating changes in different functional groups present, thus coins the interaction among artemisinin and iron oxide nanoparticles leading to the formation of ART-IONPs

Functional groups	Artemisinin (ART)	IONPs	ART-IONPs	Reference
Fe–O	—	537.56	537.56	35
CH_2_ rocking vibrations	632.74	—	—	39
	685.53			
C–H of aromatic group	822.30	—	822.30	36
	861.49		861.49	
C–C stretching	974.27	—	974.27	37
C–O–C asymmetrical stretching vibrations	1104.64	1104.64	1104.64	38
	1182.22		1182.22	
C–O vibration	1379.78	—	1379.78	40
C=O stretching	1439.76	—	1439.76	41
Free carboxylic acid	—	1633.32	1633.32	42
–C(=O)–O lactone bond	1731.70	—	1731.70	43
C–H stretching vibrations (alkanes)	2834.65	—	2834.65	36
CH_2_ stretching vibrations	2901.84	—	2901.84	44
OH stretching vibrations	—	—	3300.15	43

### 3.3 XRD analysis

X-ray diffraction (XRD) analysis (Fig. 1f) was performed to investigate the crystallinity of the synthesized MNPs, ART, and ART-MNPs. The XRD pattern of MNPs displayed characteristic peaks at 2*θ* values of 30°, 35°, 43°, 52°, 57°, and 62° (Fig. 1f). These peaks correspond to the crystal planes (111), (220), (311), (400), (422), (511), and (440) of magnetite (Fe_3_O_4_), as confirmed by their match with the reference pattern (ICDD number 00-003-0863). Notably, the XRD pattern of ART-MNPs exhibited similar peaks for iron oxide at the same positions, indicating the successful incorporation of Fe_3_O_4_ into the ART-MNPs structure.

Furthermore, the XRD pattern of ART alone revealed distinct peaks at 2*θ* values of 11.96°, 12.24°, 14.96°, 22.4°, 24.12°, and 38.56° (Fig. 1f).^45,46^ These sharp peaks suggest the crystalline nature of ART, which is crucial for maintaining its therapeutic properties. Interestingly, the Scherrer equation (eqn (1)) applied to the XRD data of ART-MNPs estimated a crystallite size of 18.53 nm. This finding suggests that the ART-MNPs possess a relatively small crystalline structure, potentially influencing their interaction with biological systems.

X-ray diffraction (XRD) analysis (Fig. 1e) was performed to investigate the crystallinity of the synthesized iron oxide nanoparticles (IONPs), artemisinin (ART), and artemisinin-iron oxide nanoparticles (ART-IONPs). The XRD pattern of IONPs displayed characteristic peaks at 2*θ* values of 30°, 35°, 43°, 52°, 57°, and 62° (Fig. 1e). These peaks correspond to the crystal planes (220), (311), (400), (422), (511), and (440) of magnetite (Fe_3_O_4_), as confirmed by their match with the reference pattern (ICDD number 00-003-0863). Notably, the XRD pattern of ART-IONPs exhibited similar peaks for iron oxide at the same positions, indicating the successful incorporation of Fe_3_O_4_ into the ART-IONPs structure.

Furthermore, the XRD pattern of ART alone revealed distinct peaks at 2*θ* values of 11.96°, 12.24°, 14.96°, 22.4°, 24.12°, and 38.56° (Fig. 1e). These sharp peaks suggest the crystalline nature of ART, which is crucial for maintaining its therapeutic properties. Interestingly, the Scherrer equation (eqn (1)) applied to the XRD data of ART-IONPs estimated a crystallite size of 8.51 nm. This finding suggests that the ART-IONPs possess a relatively small crystalline structure, potentially influencing their interaction with biological systems. Artemisinin was found complete crystalline in its XRD patterns, having strong diffraction peaks at 2*θ* of 10.92°, 11.96° and 12.24°, 14.96°, 22.4°, 24.12° and 38.56° respectively (Fig. 5). Artemisinin was found complete crystalline in its XRD patterns, having strong diffraction peaks at 2*θ* of 10.92°, 11.96° and 12.24°, 14.96°, 22.4°, 24.12° and 38.56° respectively (Fig. 5).

### 3.4 Hyperthermia study

**Fig. 2:**
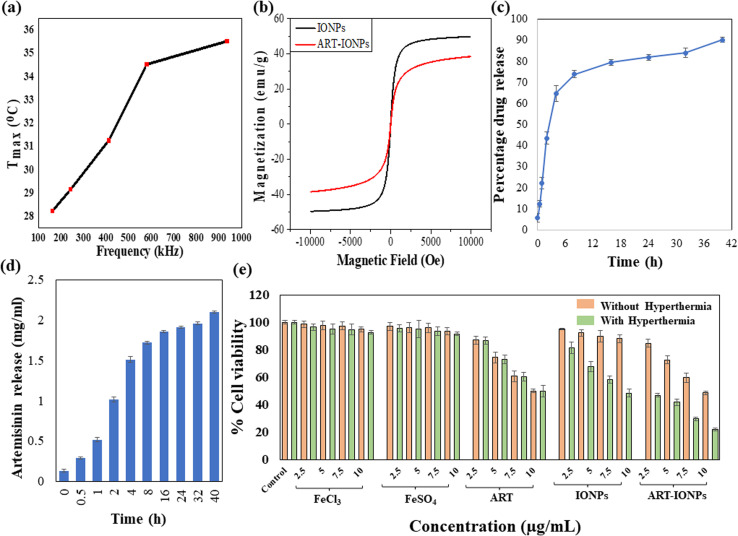
Assessment of hyperthermia sensitivity by ART-MNPs (a) it demonstrates the heating capacity of ART-MNPs at various frequencies under a constant magnetic field of 10 mT. It displays the increase in temperature, *i.e.*, 28.25 °C to 29.14 °C, 31.27 °C, 34.50 °C and 35.53 °C at diverse frequencies: 161.9 kHz, 242.4 kHz, 411.1 kHz, 580.2 kHz, and 935.3 kHz, respectively. (b) Magnetization of MNPS and ART-MNPs were recorded at r.t. (c) The graph presents the drug release studies performed for assessing the percentage of ART released from ART-MNPs at physiological pH (d) it illustrates the release rate of the drug in mg mL^−1^ at pH 7 per hour (e) the cytotoxic activity on AGS cell line was assessed using the MTT under both cases; with and without hyperthermia. It depicts the administration of treatment to AGS cells with control, including FeCl_3_ and FeSo_4_, ART, MNPS, and ART-MNPs at various concentrations of 2.5 μg mL^−1^, 5 μg mL^−1^, 7.5 μg mL^−1^, and 10 μg mL^−1^ for each sample, both in the absence and presence of hyperthermia treatment. The data were plotted as the mean of three separate experiments.

To comprehend the heating capacity of the synthesized nanoformulations under magnetic hyperthermia, temperature *vs.* time profiles were observed under different frequencies, whereas field strength and exposure time were kept constant. Each sample was exposed to hyperthermia treatment at each frequency for 10 minutes, as the hyperthermia temperature was achieved with this exposure time, beyond which overheating took place. After the hyperthermia treatment, an increase in temperature was observed from 28.25 °C to 29.14 °C, 31.27 °C, 34.50 °C, and 35.53 °C as the frequency increased from 161.9 kHz to 242.4 kHz, 411.1 kHz, 580.2 kHz, and 935.3 kHz, respectively. This trend is illustrated in Fig. 2a as a temperature (*T*_max_) *vs.* frequency (kHz) plot. The observed temperature increase demonstrates the massive potential of the nanoparticles in treating cancerous tissues by using magnetic hyperthermia.

### 3.5 Magnetic properties

The magnetic properties of MNPs and ART-MNPs were evaluated through magnetization measurements. These measurements were conducted using a Lakeshore 7404 model at room temperature with a field range of ± 10 kOe. The M–H loops of MNPs and ART-MNPs are shown in Fig. 2b. From the hysteresis loops, the saturation magnetization (*M*_s_), coercivity (*H*_c_), and remanence (*M*_r_) were determined as given in Table 2. The table shows that both remanence and coercivity values are nearly zero in MNPs and ART-MNPs, which shows that nanoparticles are superparamagnetic. This implies that thermal energy is sufficient at room temperature to overcome the magnetic anisotropy energy, keeping the nanoparticles randomly oriented without an external magnetic field. Furthermore, ART-MNPs exhibit higher saturation magnetization, which makes them one of the most potent and suitable candidates for magnetic hyperthermia applications.

**Table 2:** The table indicates the various parameters of both the synthesized nanoparticles (MNPs and ART-MNPs), which include saturation magnetization (*M*_s_), remanence magnetization (*M*_rs_), coercivity (*H*_c_), and crystallite size

Parameters	Samples	
	MNPs	ART-MNPs
*M*_s_ (emu g^−1^)	50.08	39.16
*M*_rs_	0.03	0.006
*H*_c_	0.26	0.54
Crystallite size (nm)	15.55	18.53

### 3.6 Drug loading and release kinetics

The encapsulation efficiency of ART within MNPs was determined to be 77.77 ± 3.66%, indicating a successful drug loading onto the nanoparticles. To evaluate the release profile of ART from ART-MNPs, an *in vitro* drug release study was performed using a dialysis bag method at physiological pH (pH 7.0), as described in the methods section. The cumulative release of ART over time was monitored, and the results are depicted in Fig. 2c.

As observed in Fig. 2d, the drug release profile exhibited a biphasic pattern. An initial burst release of ART was observed within the initial 8 h, followed by a sustained drug release for the next 32 h. This burst effect could be attributed to the release of ART molecules loosely adsorbed on the surface of the MNPs. The sustained release phase likely involves the slower diffusion of encapsulated ART from the inner matrix of the nanoparticles. Notably, the total cumulative ART release after 40 h reached 90.11 ± 4.12%, demonstrating the efficient drug release from the ART-MNPs. This combination of high encapsulation efficiency and controlled release highlights the efficacy of ART-MNPs as a promising drug delivery system.

Moreover, the drug release studies were carried out at gastric pH (3) and tumor pH (5.5). From the gastric pH data, it was observed that there was a burst release in the initial 8 h with nearly half of the drug (52.06 ± 2.08%) being released in the initial 2 h only, followed by a sustained drug release pattern for next 32 h with the total drug being released to be 82.52 ± 3.41% after 40 h. A similar biphasic pattern was obtained for the drug release at tumor pH, wherein the initial 8 h, 60.16 ± 1.25% drug was released, and the total drug release at the end of 40 h was found to be 78.62 ± 0.25%. The reason behind this initial burst release pattern is the release of artemisinin adsorbed on the surface of iron oxide nanoparticles followed by the slow release of drug encapsulated inside the particles. Further, in the gastric and tumor pH, which falls in the acidic range, there occurs the degradation of iron oxide nanoparticles, eventually causing the non-enzymatic cleavage of endoperoxide bridge of released artemisinin, which will further kill the cancer cells by generating organic radicals.^47^ The drug release graphs for gastric and tumor pH are represented in Fig S2.†

### 3.7 *In vitro* cell line studies with and without hyperthermia

For determining the toxicity of ART-MNPs on the AGS (gastric cancer), an MTT assay was performed under physiological conditions followed by calculating the cytotoxicity for FeCl_3_ and FeSO_4_ salt solutions, artemisinin, MNPs, and ART-MNPs at four concentrations: 2.5 μg mL^−1^, 5 μg mL^−1^, 7.5 μg mL^−1^, and 10 μg mL^−1^. Apart from the control, all the concentrations were tested on three independent sets, with and without hyperthermia treatment, to determine the effectiveness of the formulations. In the MTT assay performed without hyperthermia treatment, cell viability was measured relative to control, which was set at 100%. The results revealed several key points.

Cells after treatment with FeCl_3_ show % viability of 98.51 ± 2.35% at 2.5 μg mL^−1^, 97.71 ± 3.21% at 5 μg mL^−1^ 97.30 ± 3.02% at 7.5 μg mL^−1^, and 94.94 ± 1.80% at 10 μg mL^−1^. Similarly, cells treated with FeSO_4_ showed viability of 96.88 ± 2.69% at 2.5 μg mL^−1^, 96.01 ± 3.78% at 5 μg mL^−1^, 95.88 ± 3.31% at 7.5 μg mL^−1^ and 93.37 ± 2.59% at 10 μg mL^−1^. Cells treated with ART showed viability of 85.01 ± 2.97% at 2.5 μg mL^−1^, 74.31 ± 3.95% at 5 μg mL^−1^, 60.67 ± 3.83% at 7.5 μg mL^−1^, and 49.91 ± 1.45% at 10 μg mL^−1^. In MNPs treated cells, viability was 95.01 ± 0.6% at 2.5 μg mL^−1^, 92.24 ± 2.41% at 5 μg mL^−1^, 89.89 ± 4.15% at 7.5 μg mL^−1^ and 88.05 ± 2.78% at 10 μg mL^−1^. Finally, cells treated with ART-MNPs showed levels of viability as 84.73 ± 2.83% at 2.5 μg mL^−1^, 72.62 ± 3.07% at 5 μg mL^−1^, 59.90 ± 3.27% at 7.5 μg mL^−1^ and 48.49 ± 1.3% at 10 μg mL^−1^ concentration.

The statistical significance of data was determined by calculating *p* values for the MTT assay without hyperthermia, as represented in Table S1.† Cells treated with samples were incubated and subjected to alternating magnetic fields using a hyperthermia instrument for the hyperthermia treatment. An MTT assay was then conducted to assess cell viability. The gastric cancer cells, after treatment, exhibited a significant decrease in cell viability when compared with the control group. For FeCl_3_ salt solution-treated cells, viability was 96.45 ± 2.40% at 2.5 μg mL^−1^, 95.12 ± 3.56% at 5 μg mL^−1^, 94.63 ± 3.91% at 7.5 μg mL^−1^ and 92.61 ± 1.10% at 10 μg mL^−1^. When cells were treated with FeSO_4_ salt solution, the viability was 95.61 ± 2.45% at 2.5 μg mL^−1^, 95.22 ± 6.28% at 5 μg mL^−1^, 93.49 ± 3.30% at 7.5 μg mL^−1^ and 91.57 ± 1.23% at 10 μg mL^−1^. For ART-treated cells, the viability was 81.55 ± 2.65%, 73.15 ± 3.15%, 60.39 ± 3.05%, 49.75 ± 4.10% for the concentrations 2.5 μg mL^−1^, 5 μg mL^−1^, 7.5 μg mL^−1^ and 10 μg mL^−1^, respectively. Cells treated with MNPs, showed viability of 81.26 ± 4.23% for 2.5 μg mL^−1^, 67.78 ± 3.64% at 5 μg mL^−1^, 58.07 ± 2.75% at 7.5 μg mL^−1^ and 48.42 ± 2.74% at 10 μg mL^−1^. Lastly, ART-MNPs resulted in cell viability of 46.70 ± 1.38%, 41.67 ± 2.52%, 29.50 ± 1.20%, 21.28 ± 1.16% for 2.5 μg mL^−1^, 5 μg mL^−1^, 7.5 μg mL^−1^ and 10 μg mL^−1^, respectively.

These results indicate the cytotoxicity of ART-MNPs against AGS cells, suggesting potential for further exploration of anti-cancer potential as given in Fig. 2e. The statistical significance of the results was determined by calculating *p* values for the MTT assay with hyperthermia, as represented in Table S2.†

### 3.8 Scratch assay for anti-cancer activity

**Fig. 3:**
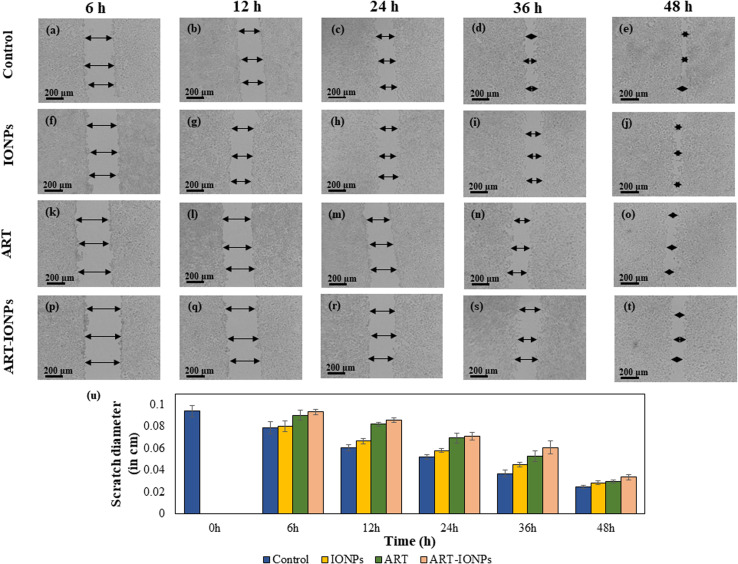
The figure depicts the anti-cancer impact of ART-MNPs on the AGS cell line through a scratch assay (a) to (e) are control cells, MNPS treated cells at a fixed concentration of 10 μg mL^−1^ (f) to (j), ART-treated cells (k) to (o) with a concentration of 10 μg mL^−1^, cells treated with ART-MNPs (p) to (t) with 10 μg mL^−1^ concentration (u) the data in graph illustrating the comparison of alterations in scratch diameter after treatment with ART, MNPS, and ART-MNPs including control after 6 h, 12 h, 24 h, 36 h, and 48 h.

The scratch assay (Fig. 3a–t) was used to evaluate the anti-proliferative effects of ART-MNPs on AGS cells. Cells were treated with 10 μg mL^−1^ of ART, MNPs, or ART-MNPs. Scratch closure was quantified by measuring the decrease in scratch diameter at three points per well, with the mean value reported. Control cells displayed a gradual scratch closure, reaching 73.71 ± 0.12% after 48 h (Fig. 3u). MNPs exhibited weaker anti-proliferative activity (52.16 ± 0.21% at 36 h, 71.98 ± 0.63% at 48 h), while ART treatment showed a moderate effect (43.54 ± 0.50% at 36 h, 68.32 ± 0.12% at 48 h). ART-MNPs demonstrated the most significant inhibition of cell migration (0.66 ± 0.24% at 6 h, 64.23 ± 0.27% at 48 h). This superior efficacy suggests a synergistic effect between ART and MNPs in the ART-MNPs formulation (detailed statistical analysis in Table S3†).

### 3.9 Detection of autophagic vacuoles by MDC

**Fig. 4:**
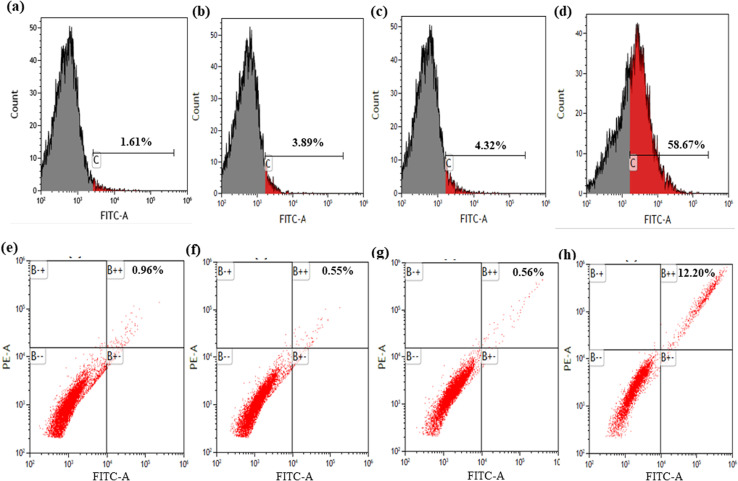
The figure shows the percentage of autophagy in cells using MDC (monodansylcadaverine) dye (a) control cells, which have 1.61% apoptotic cells; (b) MNPS treated cells, which showed 3.89% apoptotic cells, (c) in ART-treated cells, the percentage of apoptotic cells is 4.32% and (d) in ART-MNPs treated cells, the percentage of apoptotic cells was found to be the highest as 58.65%. Percentage of autophagy in cells using A.O. (acridine orange) dye (e) in control cells, the percentage of apoptotic cells came out to be 0.96%, (f) in mnps treated cells, the percentage of apoptotic cells is 0.55%, (g) in art-treated cells, the percentage of apoptotic cells is 0.56% and (h) in ART-MNPs treated cells showed the highest percentage apoptotic cells were found to be 12.20%.

To assess the alteration of the extent of cellular autophagy effects of the synthesized formulations, AGS cells were treated and analyzed using flow cytometry. As shown in Fig. 4a, control cells exhibited a basal level of apoptosis (1.61%). Treatment with MNPs (Fig. 4b) and ART (Fig. 4c) resulted in moderate increases in apoptotic cells (3.89% and 4.32%, respectively). Notably, ART-MNPs (Fig. 4d) induced a significantly higher percentage of apoptotic cells (58.65%), suggesting their enhanced anti-cancer potential.

### 3.10 Detection of autophagic vacuoles by A.O.

Flow cytometry analysis was employed to quantify the potential impact of the synthesized formulation on autophagy in AGS cells (Fig. 4e). Control cells displayed a basal level of apoptosis (0.96%). Treatment with MNPs (Fig. 4f) and ART (Fig. 4g) did not significantly alter the apoptotic cell population (0.55% and 0.56%, respectively). Interestingly, ART-MNPs (Fig. 4h) induced a significantly higher percentage of apoptotic cells (12.20%), suggesting their potential to induce cell death.

### 3.11 Anti-bacterial effect of ART-MNPs on *H. pylori*

#### 3.11.1 Anti-bacterial activity by determining the MIC value

**Fig. 5:**
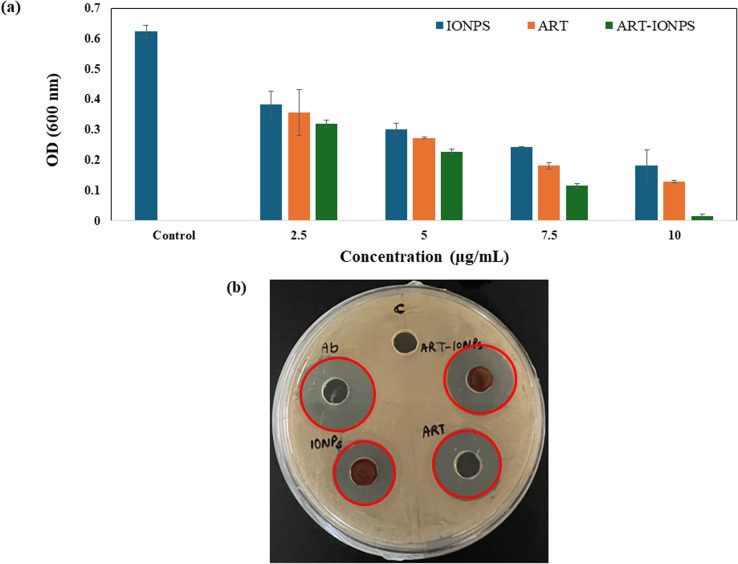
Monitoring anti-*H. Pylori* using ART-MNPs (a) the graph represents the MIC values, and it comes out to be 10 μg mL^−1^ for ART-MNPs (b) the figure shows the zone of inhibition for *H. Pylori* that is ∼9.5 mm, ∼10.5 mm, and ∼11.5 mm when treated with MNPS, ART, and ART-MNPs respectively.

Anti-bacterial activity was assessed by following the broth dilution method, and the MIC value was established. The MIC value for ART-MNPs was determined to be 10 μg mL^−1^, see Fig. 5a. The enhanced anti-bacterial activity is likely due to the influx of synthesized nano-formulations being easy for Gram-negative bacteria's cell wall, which has a unique outer membrane. These nanoparticles, in conjunction with artemisinin, are responsible for the generation of ROS, which eventually kills the bacteria. The statistical analysis is presented in Table S4.†

#### 3.11.2 Anti-bacterial activity by agar well diffusion assay

The anti-bacterial activity was further confirmed by the agar well diffusion method. The zone of inhibition for *H. pylori* after treatment with MNPS, ART, and ART-MNPs were ∼9.5 mm, ∼10.5 mm, and ∼11.5 mm, respectively, indicating the superior anti-bacterial activity and zone of inhibition of ART-MNPs in comparison to MNPs and ART alone (Fig. 5b).

### 3.12 Combination index of iron and insulin

The combination index (CI) was calculated to quantify the interaction between ART and MNPs. *Dm* values, derived from Fig. S3a (ART) and S3b (MNPs),† were used in the CI calculation. As shown in Table 3, the CI values for all tested combinations were below 1, indicating a synergistic effect. This finding aligns with the *in vitro* cell viability and scratch assay results (see previous sections, 3.7 and 3.8), suggesting that ART and MNPs work together to enhance their anti-cancer activity.

**Table 3:** The table presents the combination index (CI) data derived from varying concentrations of ART and IONPs in combination to assess whether the two drugs demonstrate synergistic or antagonistic effects on cell viability. The values obtained are less than 1, indicating a synergistic effect between ART and IONPs

Concentration of Fe salt and insulin	(*Dx*)_1_ (Fe salt) = *Dm*[*fa*/*fu*]^1/*m*^	(*Dx*)_1_ (insulin) = *Dm*[*fa*/*fu*]^1/*m*^	CI = (*D*)_1_/(*Dx*)_1_ + (*D*)_2_/(*Dx*)_2_
2.5 μg mL^−1^	99.28705	112.0501	0.047491
5 μg mL^−1^	111.495	128.556	0.083739
7.5 μg mL^−1^	130.6199	147.8165	0.108157
10 μg mL^−1^	158.1126	180.3481	0.118694

## 4. Conclusions

This study has successfully explored the role of artemisinin-magnetic iron oxide nanoparticles as a novel therapeutic strategy for gastric cancer. By combining the anti-cancer properties of ART with the hyperthermic capabilities of MNPs, ART-MNPs offer a synergistic approach for targeted cancer cell eradication. Our findings demonstrate the successful development of ART-MNPs with high encapsulation efficiency and a biphasic drug release profile. This indicates effective drug loading and the potential for rapid delivery upon reaching the target site, followed by a sustained therapeutic effect. Most importantly, ART-MNPs exhibited significantly enhanced anti-proliferative activity compared to individual treatments with ART or MNPs alone. This synergistic effect was evident in the scratch assay results, suggesting that the combination therapy disrupts multiple cellular processes, potentially leading to more efficient cancer cell eradication.

Mechanistically, ART is known to generate reactive oxygen species (ROS) *via* interaction with ferrous iron, leading to mitochondrial damage and cell death. MNPs, when exposed to an external magnetic field, can generate localized heat (hyperthermia), further sensitizing cancer cells to the cytotoxic effects of ART. This synergistic action highlights the potential of ART-MNPs to overcome a significant challenge in cancer treatment: hyperthermia resistance. Additionally, ART-MNPs also demonstrated anti-*H. pylori* activity, which would further enhance the efficacy of the treatment.

In conclusion, this study has demonstrated the promising potential of ART-MNPs for targeted and synergistic therapy against gastric cancer. Their ability to combine the anti-cancer properties of ART with the hyperthermic effects of MNPs opens new avenues for developing more effective treatment strategies. Further research on overcoming hyperthermia resistance through autophagy modulation and exploring combination therapies with other modalities is warranted to fully translate the potential of ART-MNPs into clinical practice, ultimately improving the lives of cancer patients.

## Data availability

The data will be made available upon request.

## Author contributions

Conception and design of the study: K. Attri, B. N. Chudasama, R. L. Mahajan, D. Choudhury. Experimentation: K. Attri. Analysis/interpretation of data: K. Attri, D. Choudhury. Drafting the Manuscript: K. Attri. Fund acquisition: D. Choudhury. Critical reviewing of manuscript: B. N. Chudasama, R. L. Mahajan, D. Choudhury. Approval of the final manuscript: K. Attri, B. N. Chudasama, R. L. Mahajan, D. Choudhury.

## Conflicts of interest

There are no conflicts to declare.

## Supplementary Material

RA-014-D4RA05611F-s001
