# Supplementary material for: Perturbation of hyperthermia resistance in gastric cancer by hyperstimulation of autophagy using artemisinin-protected iron-oxide nanoparticles
Source: RSC Adv. 2024 Oct 29;14(47):34565–77. doi: 10.1039/d4ra05611f (PMC11520311; doi:10.1039/d4ra05611f)
Supplement: RA-014-D4RA05611F-s001 [file RA-014-D4RA05611F-s001.pdf]

Supplementary Information (SI) for RSC Advances.
This journal is © The Royal Society of Chemistry 2024

Perturbation of Hyperthermia Resistance in Gastric Cancer by Hyperstimulation of Autophagy using Artemisinin-protected Iron-oxide Nanoparticles

Komal Attri[1,4], Bhupendra Chudasama[2,4*], Roop L. Mahajan[3,4*], Diptiman Choudhury[1,4*]

[1]Department of Chemistry and Biochemistry, Thapar Institute of Engineering and Technology, Patiala-147004, Punjab, India.

[2]Department of Physics and Material Sciences, Thapar Institute of Engineering and Technology, Patiala-147004, Punjab, India

[3]Department of Mechanical Engineering, Department of Materials Science & Engineering, Virginia Tech, Blacksburg, VA 24061, United States

[4]TIET-VT Centre of Excellence for Emerging Materials, Thapar Institute of Engineering and Technology, Patiala-147004 Punjab, India.

*Corresponding E-mail: diptiman@thapar.edu, mahajanr@vt.edu, bnchudasama@thapar.edu

*Corresponding Phone: +91-8196949843 / +1-5402312597 / +91-9781966136

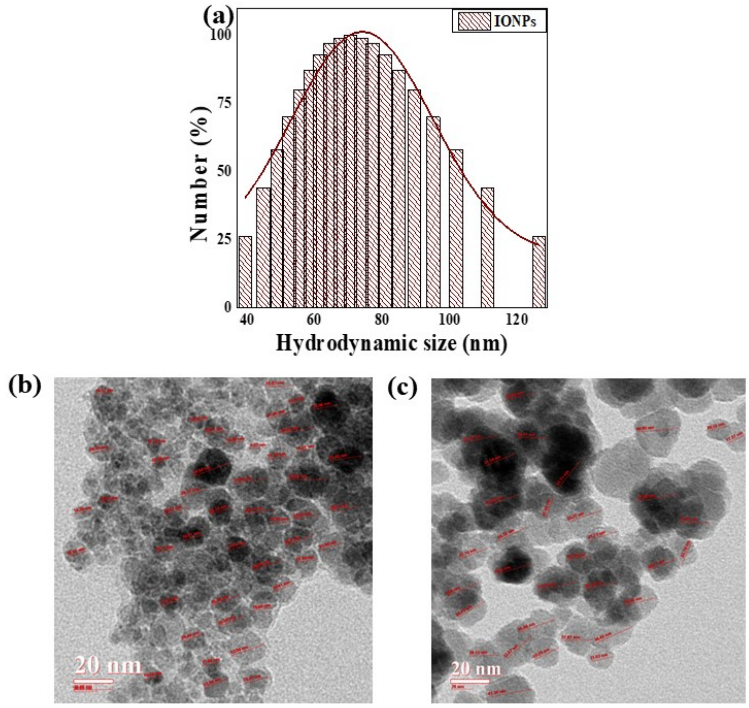

Figure S1: DLS and HRTEM figures of IONPs and ART-MNPs (a) It represents the DLS graph of IONPs having a size of 74 nm (b) HR-TEM image showing the size measurement of each particle of IONPs (c) HR-TEM image showing the size measurement of each particle of ART-MNPs.

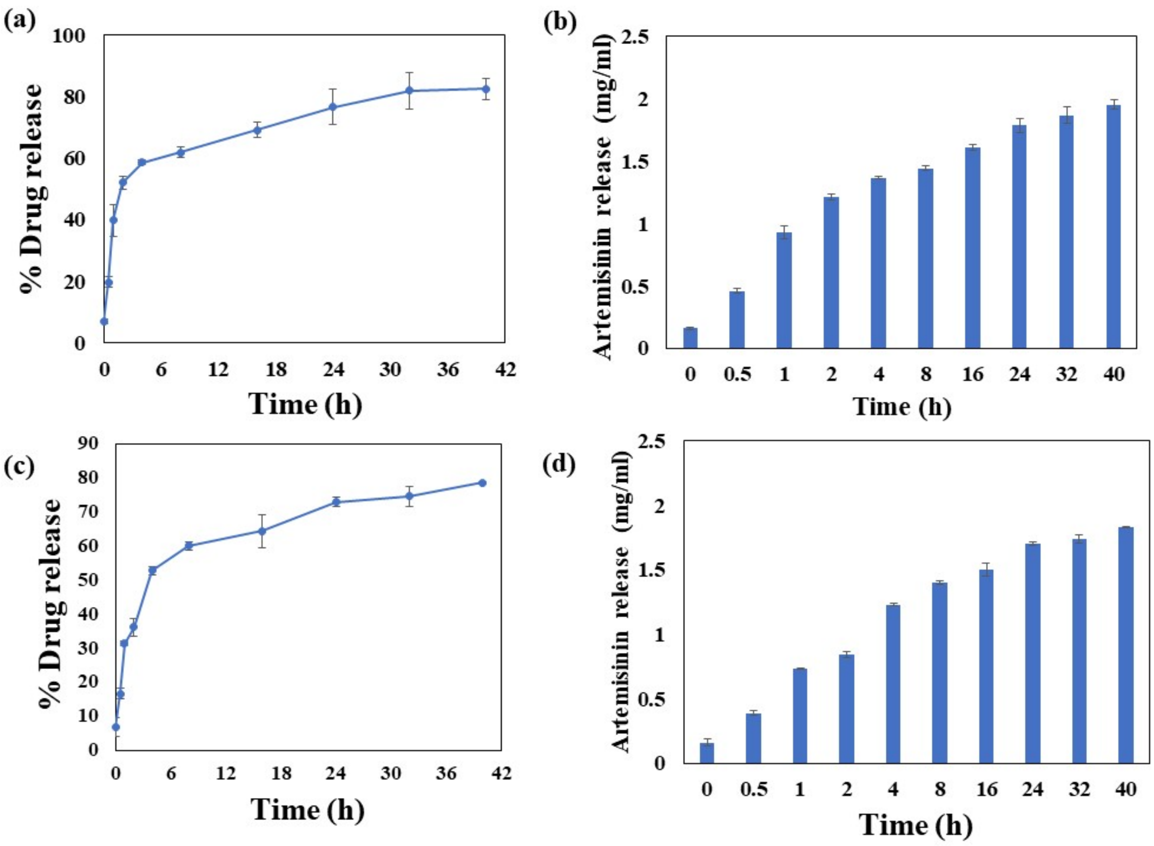

Figure S2: Release kinetics of Artemisinin in gastric and tumor pH. (a) The graph presents the drug release studies performed for assessing the percentage of ART released from ART-MNPs at gastric pH (3) (b) It illustrates the release rate of the drug in mg/ml at pH 3 per hour (c) The graph depicts the drug release studies conducted to evaluate the percentage of ART released from ART-MNPs at tumor pH (5.5) (d) It depicts the drug release rate in mg/ml per hour at tumor pH (5.5).

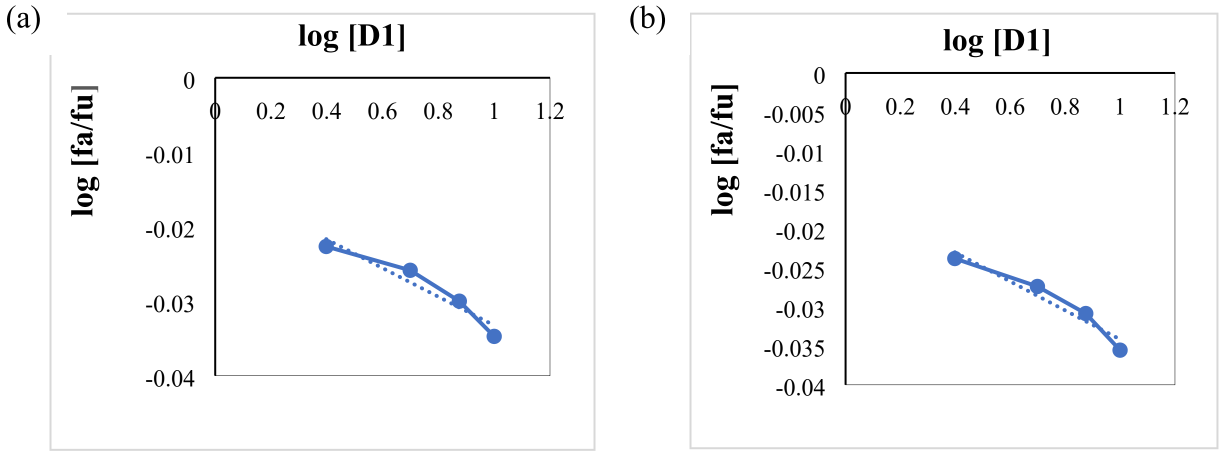

Figure S3: Median plots of (a) ART **and (b)** MNPs for finding the y-intercept and m values to calculate Dm and determine the combination index of ART and MNPs.

Table S1 shows the p values calculated for % variation in cell viability after treatment with 2.5, 5, 7.5, and 10 µg/mL of ART, MNPs, and ART-MNPs without hyperthermia. The statistical significance of data is considered when the $p < 0.05$

**P value for checking the statistical significance of data (Without Hyperthermia)**			
**Concentration**	**ART**	**MNPs**	**ART-MNPs**
**2.5µg/mL**	0.002413	0.002773	0.00112
**5µg/mL**	0.000477	0.008284	0.000152
**7.5 µg/mL**	8.06E-05	0.017229	0.000152
**10 µg/mL**	1.39E-06	0.002665	9.43E-07

Table S2 shows the p values calculated for % variation in cell viability after treatment with 2.5, 5, 7.5, and 10 µg/mL of ART, MNPs, and ART-MNPs with hyperthermia. The statistical significance of data is considered when the $p < 0.05$

**P value for checking the statistical significance of data (With Hyperthermia)**			
**Concentration**	**ART**	**MNPs**	**ART-MNPs**
**2.5µg/mL**	0.009367	0.009526	1.4201E-05
**5µg/mL**	0.000869	0.000836	3.04E-05
**7.5 µg/mL**	0.000336	0.000139	4.13E-06
**10 µg/mL**	0.000212	6.07E-05	2.65E-06

Table S3 shows the p values calculated for % variation in scratch diameter after treatment with 10 µg/ml of ART, MNPs, and ART-MNPs. The statistical significance of data is considered when the $p < 0.05$.

**P value for checking the statistical significance of data**			
**Time**	**ART**	**MNPs**	**ART-MNPs**
**6h**	0.054767	0.792443	0.004362
**12h**	0.000305	0.041174	0.000243
**24h**	0.004036	0.023064	0.001396
**36h**	0.01039	0.027416	0.004124
**48h**	0.00749	0.043244	0.006911

Table S4 shows the p values calculated for % variation in the absorbance value of *H. pylori* after treatment with varying concentrations (2.5, 5, 7.5, 10 µg/ml) of ART, MNPs, and ART-MNPs. The statistical significance of data is considered when the $p < 0.05$.

**P value is used to check the statistical significance of data for anti-*H. pylori* activity**			
	**ART**	**MNPs**	**ART-MNPs**
**2.5µg/mL**	0.004458	0.00133	5.46E-05
**5µg/mL**	2.09E-05	7.73E-05	1.56E-05
**7.5 µg/mL**	1.1E-05	1.5E-05	5.43E-06
**10 µg/mL**	5.46E-06	0.000213	2.48E-06
